# Macrophage-Centered Integration of Single-Cell and Bulk Transcriptomic Data Identifies CCL3, CCL4, and JUNB as Inflammatory Regulatory Signatures in Ulcerative Colitis

**DOI:** 10.3390/genes17070841

**Published:** 2026-07-22

**Authors:** Haoyang Meng, Yongliang Chen, Yongchun Chai, Sike Yu, Peiyao Ma, Ruibin Lei, Shuhan Zhou, Wenliang Lv

**Affiliations:** 1 College of Traditional Chinese Medicine, Hubei University of Chinese Medicine, Wuhan 430061, China; 2432701185@stmail.hbucm.edu.cn (H.M.); cyl@stmail.hbucm.edu.cn (Y.C.); 2432701139@stmail.hbucm.edu.cn (Y.C.); 2 School of Pharmaceutical Science and Technology, University of Chinese Academy of Sciences, Beijing 100049, China; yusike23@mails.ucas.ac.cn (S.Y.); leiruibin22@mails.ucas.ac.cn (R.L.); 3 School of Biology and Biological Engineering, South China University of Technology, Guangzhou 510006, China; mapeiyao@mail.scut.edu.cn

**Keywords:** ulcerative colitis, macrophages, single-cell RNA sequencing, transcriptomics, cell–cell communication, regulatory network

## Abstract

Objectives: This study aimed to identify and characterize macrophage-associated inflammatory regulatory signatures in ulcerative colitis (UC) by integrating bulk and single-cell transcriptomic data, and to explore their potential regulatory and pharmacological relevance. Methods: Two colonic bulk microarray datasets (GSE179285 and GSE87466) and one single-cell RNA-sequencing dataset (GSE231993) were analyzed. Differential expression analysis, area under the recovery curve-based single-cell gene-set scoring (AUCell) scoring, macrophage high-dimensional Weighted Gene Co-Expression Network Analysis (hdWGCNA), and three machine learning algorithms were combined to prioritize candidate genes. Their expression and diagnostic performance were externally validated. Macrophage trajectory analysis, cell–cell communication analysis, virtual perturbation, transcription factor activity inference, compound prediction, and molecular docking were further performed. Results: Single-cell preprocessing retained 30,737 high-quality cells, and macrophages exhibited relatively high innate immune cell barrier-related gene activity. Integrated screening identified CCL3, CCL4, JUNB, and FOS, whereas machine learning consensus retained CCL3, CCL4, and JUNB as the final signatures. These genes were consistently upregulated in UC, with validation area-under-the-curve values of 0.917, 0.958, and 0.888, respectively. Their expression varied along an inferred macrophage inflammatory state continuum, and UC showed remodeled macrophage-centered communication, including CXCL8–ACKR1 signaling. Virtual perturbation linked CCL3 and CCL4 to chemotaxis and lysosomal programs and JUNB to antigen processing and major histocompatibility complex (MHC) class II pathways; RFX5 was prioritized as a potential upstream regulator. CID11879209 was predicted as a shared candidate compound, with docking energies of −6.55, −6.31, and −4.50 kcal/mol for CCL3, CCL4, and JUNB, respectively. Conclusions: CCL3, CCL4, and JUNB constitute a macrophage-associated inflammatory signature connecting tissue-level UC dysregulation with macrophage state remodeling. These findings provide testable molecular and pharmacological hypotheses requiring further experimental and clinical validation.

## 1. Introduction

Ulcerative colitis (UC) is a relapsing inflammatory bowel disease (IBD) characterized by continuous colonic mucosal inflammation, epithelial barrier injury, crypt architectural distortion, and heterogeneous clinical behavior. Current evaluation relies on symptoms, endoscopy, histology, serum inflammatory markers, and fecal biomarkers, yet these measurements provide limited resolution of molecular heterogeneity within active mucosal inflammation [1]. Treatment has expanded from 5-aminosalicylates and corticosteroids to biologics and small-molecule agents, but durable remission, mucosal healing, and treatment positioning remain difficult across patient subgroups [2,3]. Treat-to-target strategies have improved the conceptual framework for monitoring disease control, while the therapeutic ceiling in UC drug development highlights the need for molecular signatures that capture tissue-level immune remodeling and biological heterogeneity [4,5].

Innate immune remodeling is a central component of UC mucosal pathology. Intestinal macrophages are particularly relevant because they integrate microbial recognition, phagocytosis, cytokine and chemokine release, antigen processing, epithelial/stromal interaction, tissue injury, and repair. Human intestinal macrophages have been implicated in both UC and Crohn’s disease, and cell-resolved analyses have shown that IBD lesions contain distinct myeloid and macrophage states rather than a single static macrophage phenotype [6,7]. Spatially informed and single-cell studies further indicate that macrophage state organization can be linked to local tissue topology and inflammatory niches within UC mucosa [7,8].

Bulk transcriptomic datasets provide disease-level expression signals from clinically annotated tissue samples, while single-cell RNA-seq resolves the cellular sources and state-specific distribution of these signals. Their integration is especially useful in inflamed mucosa, where observed expression differences reflect both transcriptional regulation and shifts in cellular composition. Recent reviews and multi-omics analyses in IBD emphasize that biomarker interpretation is strongest when disease-level signatures are connected with cellular context and independent validation [9,10]. Integrated UC studies have also used co-expression analysis, pathway annotation, and external validation to nominate disease-associated hub genes, illustrating the value of transparent computational prioritization in UC transcriptomics [11,12].

This study integrated public colonic bulk transcriptomic microarray data, colonic single-cell RNA-seq data, innate immune cell barrier-related genes, macrophage high-dimensional Weighted Gene Co-Expression Network Analysis (hdWGCNA), machine learning, pathway enrichment, macrophage trajectory inference, cell–cell communication analysis, virtual perturbation, transcription factor activity analysis, compound prediction, and molecular docking. The aim was to define macrophage-associated inflammatory regulatory signatures in UC and to interpret how these signatures connect tissue-level disease dysregulation with macrophage state remodeling and candidate intercellular and regulatory networks.

## 2. Methods

### 2.1. Study Design and Public Datasets

This study was designed as a retrospective integrative transcriptomic analysis of publicly available human colonic datasets. The analytical workflow combined bulk transcriptomic microarray discovery, external microarray validation, and single-cell RNA-seq-based cell-type interpretation.

Datasets were retrieved from the Gene Expression Omnibus (GEO) (https://www.ncbi.nlm.nih.gov/geo/, accessed on 1 April 2026). GSE179285 was used as the discovery dataset and included 23 inflamed UC colon samples and 23 healthy control colon samples profiled on GPL6480 [13]. GSE87466 was used as the external validation dataset and included 87 UC samples and 21 healthy control samples profiled on GPL13158 [14]. GSE231993 was used as the single-cell RNA-seq dataset and included inflamed UC and healthy control colon samples from four patients with UC and four healthy controls profiled on GPL18573 [8].

Innate immune cell barrier-related gene (IICBRG) were compiled from the Kyoto Encyclopedia of Genes and Genomes (KEGG) (https://www.genome.jp/kegg/, accessed on 1 April 2026), ImmPort (https://www.immport.org/shared/home, accessed on 1 April 2026), Molecular Signatures Database (MSigDB) (https://www.gsea-msigdb.org/gsea/msigdb, accessed on 1 April 2026), and InnateDB (https://www.innatedb.com/), yielding 1356 nonredundant genes after deduplication [15,16,17,18].

### 2.2. Single-Cell RNA-Seq Preprocessing and Annotation

Single-cell RNA-seq data from GSE231993 were processed using Seurat v5.1.0 (https://satijalab.org/seurat/, accessed on 15 April 2026) [19]. Raw count matrices were read using Read10X and converted into Seurat objects. Cells were retained when nFeature_RNA was greater than 200 and less than 3000, percent.mt was less than 25, and nCount_RNA was less than 25,000. The dataset contained 39,904 cells and 22,471 genes before quality control and 30,737 cells and 22,471 genes after filtering. Data were normalized using LogNormalize, the top 2000 highly variable genes were selected with FindVariableFeatures, and dimensionality reduction was performed by principal component analysis (PCA) followed by uniform manifold approximation and projection (UMAP). Twenty principal components and a clustering resolution of 0.6 were used for global clustering.

Major cell types were manually annotated according to canonical and dataset-reported marker genes. EPCAM marked epithelial cells, DCN marked smooth muscle cells, CD31 marked endothelial cells, SPARC marked fibroblasts, CD3 marked T cells, CD79B marked B cells, CD68 marked macrophages, GNLY marked natural killer cells, and CPA3 marked mast cells.

### 2.3. IICBRG Scoring and Macrophage HdWGCNA

IICBRG activity was evaluated at the single-cell level using area under the recovery curve-based single-cell gene-set scoring (AUCell) v1.22.0 (https://bioconductor.org/packages/AUCell/, accessed on 15 April 2026) [20]. Cell-type-level area under the curve (AUC) score distributions were compared across annotated cell populations and then within innate immune candidate populations. Macrophages showed higher IICBRG activity than mast cells and were selected for downstream macrophage-centered analysis.

Macrophage-associated co-expression analysis was performed using hdWGCNA v0.4.00 (https://smorabit.github.io/hdWGCNA/, accessed on 15 April 2026) [21]. Genes expressed in at least 5% of macrophages were retained. Metacells were constructed with k = 25 and max_shared = 10. Soft_power = 14 was selected for network construction. The blue module was retained for candidate gene intersection because kME-ranked hub gene visualization, UMAP module eigengene mapping, and module feature association supported its relevance within the macrophage co-expression network.

### 2.4. Bulk Differential Expression and Candidate Gene Definition

Bulk differential expression analysis was conducted in GSE179285 using limma v3.56.2 [22]. Inflamed UC colon samples were compared with healthy control colon samples. Differentially expressed genes (DEGs) were defined using |log2 fold change| > 0.5 and *p* < 0.05. Volcano plots were generated with ggplot2 v3.5.1, and heatmaps were generated with ComplexHeatmap v2.16.0 [23,24]. Candidate macrophage-associated genes were obtained by intersecting UC-associated DEGs, IICBRGs, and genes from the macrophage hdWGCNA blue module using ggvenn v0.1.10.

### 2.5. Machine Learning and Transcriptomic Discrimination

Support vector machine/recursive feature elimination, random forest, and Boruta were independently applied to the candidate macrophage-associated genes in GSE179285. Support vector machine/recursive feature elimination was performed using e1071 v1.7-16 with a radial basis function kernel and fivefold cross-validation, following the support vector machine framework [25]. Random forest analysis was performed using randomForest v4.7-1.2; 1 to 100 trees were evaluated, ntree = 42 was selected according to the minimum error rate, and the top three genes ranked by feature importance were retained [26]. Boruta analysis was performed using Boruta v8.0.0 and retained confirmed features [27]. Genes retained by all three approaches were defined as the final macrophage-associated signatures.

Expression differences for the final signature genes were compared between UC and healthy control samples in the discovery and validation datasets. Transcriptomic discriminative capacity was evaluated using receiver operating characteristic curves generated with pROC v1.18.5, and area under the curve values were calculated for each gene [28]. A three-gene nomogram was constructed in the discovery dataset using rms v6.8.1. Calibration analysis was generated with regplot v1.1. The combined receiver operating characteristic curve and decision curve analysis were generated with pROC v1.18.5 and ggDCA v1.1.

### 2.6. Functional Enrichment, Localization, Trajectory, and Communication Analyses

Gene set enrichment analysis was performed for CCL3-, CCL4-, and JUNB-associated expression profiles using the MSigDB c2.cp collection (https://www.gsea-msigdb.org/gsea/msigdb/collections.jsp, accessed on 15 April 2026) [17]. Genes were ranked by Spearman correlation coefficients between each signature gene and all other genes, and pathways from c2.cp.v2022.1.Hs.symbols.gmt were considered significant at |NES| > 1 and *p* < 0.05. Enrichment analysis was implemented with clusterProfiler [29]. Chromosomal localization was obtained from gene annotation resources, and subcellular localization was evaluated using RNALocate.

Macrophages were extracted from the annotated single-cell dataset and re-clustered into transcriptionally distinct macrophage states. These states were annotated as resident, proliferating, and infiltrating macrophage states according to marker gene patterns. Pseudotime analysis was conducted with Monocle (http://cole-trapnell-lab.github.io/monocle-release/, accessed on 15 April 2026) to evaluate CCL3, CCL4, and JUNB expression along the inferred macrophage transcriptional state continuum [30].

Cell–cell communication was inferred using CellChat v1.6.1 (https://github.com/jinworks/CellChat, accessed on 15 April 2026) [31]. UC and healthy control cells were analyzed separately to compare macrophage-centered incoming and outgoing communication networks.

### 2.7. Virtual Perturbation, Transcription Factor Activity, Compound Prediction, and Docking

Virtual perturbation of CCL3, CCL4, and JUNB in macrophages was conducted with scTenifoldKnk (https://github.com/cailab-tamu/scTenifoldKnk, accessed on 15 April 2026) [32], and protein–protein interaction (PPI) networks were constructed using Search Tool for the Retrieval of Interacting Genes/Proteins (STRING) (https://string-db.org/, accessed on 15 April 2026) [33]. Transcription factor activity was inferred with DoRothEA to identify potential upstream regulatory components associated with the macrophage signatures [34].

Candidate compounds targeting the final signature genes were predicted using GraphBAN (https://github.com/peizhenbai/GraphBAN, accessed on 30 April 2026) and the ZINC-250K compound library (https://figshare.com/articles/dataset/ZINC_250K_data_sets/17122427, accessed on 30 April 2026) [35,36]. Protein sequences were retrieved from UniProt (https://www.uniprot.org/, accessed on 30 April 2026), and compound structures were checked through PubChem when CID identifiers were available.

Protein structures were obtained from RCSB PDB (https://www.rcsb.org/, accessed on 30 April 2026), AlphaFold DB (https://alphafold.ebi.ac.uk/, accessed on 30 April 2026), and PubChem (https://pubchem.ncbi.nlm.nih.gov/, accessed on 30 April 2026). Molecular docking was performed using AutoDock v1.5.7 to evaluate predicted interactions between CID11879209 and CCL3, CCL4, or JUNB [37].

### 2.8. Statistical Analysis

Unless otherwise specified, two-group comparisons used the Wilcoxon rank-sum test, and *p* < 0.05 was considered statistically significant. Receiver operating characteristic curves were summarized by area under the curve. Pathway-level enrichment used |NES| > 1 and *p* < 0.05.

## 3. Results

### 3.1. Single-Cell Preprocessing Established a Cellular Framework for UC Mucosal Interpretation

**Figure 1:**
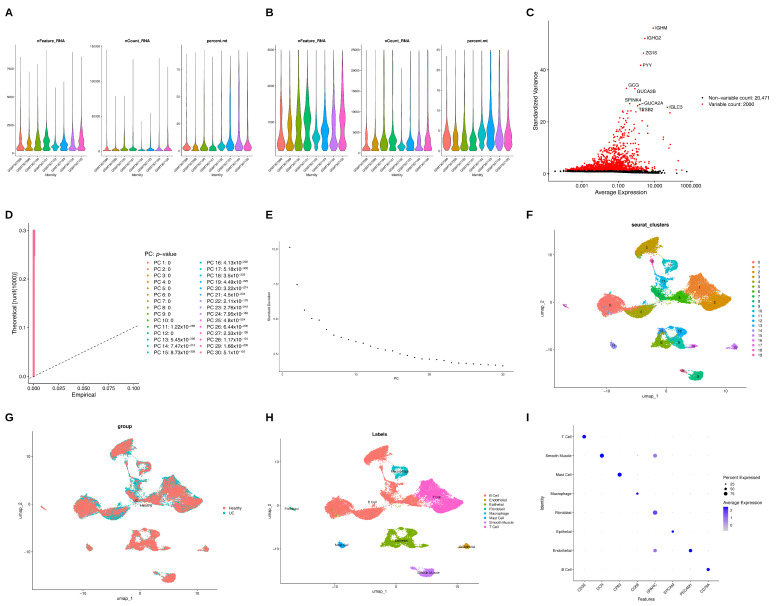
Single-cell RNA-seq preprocessing and cell-type annotation. (**A**,**B**) Pre- and post-quality control distributions of nFeature_RNA, nCount_RNA, and percent.mt. (**C**) Highly variable genes. (**D**) PCA permutation plot. (**E**) Elbow plot. (**F**) UMAP clusters. (**G**) Disease-group UMAP plot. (**H**) Annotated cell-type UMAP plot. (**I**) Marker gene dot plot.

The GSE231993 single-cell dataset was processed to construct a cell-resolved map of UC and healthy control colonic mucosa. Initial quality control distributions showed broad variation in detected gene number, total molecular counts, and mitochondrial transcript percentage, indicating the need to remove low-quality or extreme cells before downstream interpretation. After applying thresholds for nFeature_RNA, nCount_RNA, and percent.mt, 30,737 cells and 22,471 genes were retained from the initial 39,904 cells and 22,471 genes. This retained cell set provided sufficient cellular breadth to evaluate both structural mucosal compartments and immune cell populations (Figure 1A,B).

Normalization and highly variable gene selection reduced technical differences related to sequencing depth and focused the analysis on genes with strong cell-to-cell variation. PCA and the elbow plot pattern supported the use of 20 principal components for graph-based clustering. UMAP visualization resolved 20 clusters, with UC and healthy control cells occupying partially shared and partially disease-enriched regions of the embedding (Figure 1C–G). This distribution is consistent with a mucosal dataset in which disease status modifies the abundance and transcriptional state of multiple cell types rather than producing complete separation of all cells by diagnosis; such mixed but disease-shifted cellular organization has also been emphasized in single-cell and spatial analyses of IBD [7,8,9].

Manual marker-based annotation identified epithelial cells, smooth muscle cells, endothelial cells, fibroblasts, T cells, B cells, macrophages, natural killer cells, and mast cells (Figure 1H,I). The marker-gene dot plot supported these assignments by showing expected enrichment of EPCAM in epithelial cells, DCN in smooth muscle cells, CD31 in endothelial cells, SPARC in fibroblasts, CD3 in T cells, CD79B in B cells, CD68 in macrophages, GNLY in natural killer cells, and CPA3 in mast cells. This annotation step established the cellular context required to interpret disease-associated bulk transcriptomic signals at single-cell resolution.

### 3.2. IICBRG Scoring and Macrophage HdWGCNA Defined a Macrophage-Centered Co-Expression Layer

**Figure 2:**
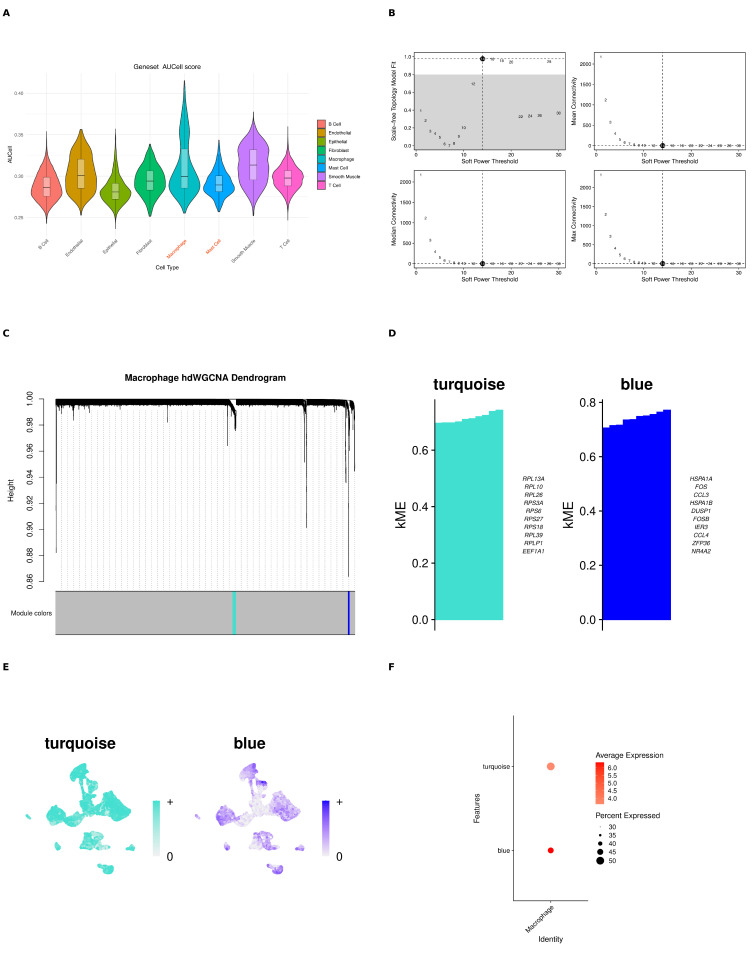
IICBRG scoring and macrophage hdWGCNA. (**A**) AUCell score distributions across annotated cell types. (**B**) Soft-thresholding power selection. The gray-colored region indicates soft-thresholding powers with a scale-free topology fit index (scale independence) below 0.8, which did not meet the predefined scale-free topology criterion. The vertical dashed line marks the selected optimal soft-thresholding power (soft_power = 14). (**C**) hdWGCNA dendrogram. (**D**) kME-ranked hub-gene visualization. (**E**) UMAP module eigengene maps. (**F**) Module feature bubble plot.

AUCell scoring was used to quantify IICBRG activity in individual cells and then compare score distributions across annotated cell types. Macrophages displayed relatively high IICBRG activity in the global cell-type comparison. Among the innate immune candidate populations explicitly represented in the annotation, macrophages showed higher IICBRG activity than mast cells (Figure 2A). This result prioritized macrophages as the key cell compartment for downstream co-expression and regulatory analysis. The selection reflected IICBRG activity within the available single-cell annotation and supported a macrophage-centered interpretation of innate immune barrier dysregulation in UC mucosa.

Macrophage hdWGCNA was then performed to identify co-expression structure within the macrophage compartment. Genes expressed in at least 5% of macrophages were retained to reduce sparsity, and metacells were constructed to stabilize network inference in single-cell data. Soft_power = 14 was selected for network construction, and two macrophage co-expression modules were detected (Figure 2B,C). The blue module was selected because kME-ranked hub gene visualization, UMAP module eigengene mapping, and module feature association supported its macrophage relevance (Figure 2D–F). The blue module contained 22 genes and was carried forward as the macrophage co-expression component for candidate gene intersection. The complete list of genes in the macrophage hdWGCNA blue module, together with their module membership (kME) values, is provided in Supplementary Materials, Additional File S1. This network layer ensured that subsequent candidates were connected to macrophage co-expression architecture rather than selected solely by tissue-level differential expression.

### 3.3. Bulk Differential Expression and Intersection Narrowed Disease-Level Dysregulation to Four Candidates

**Figure 3:**
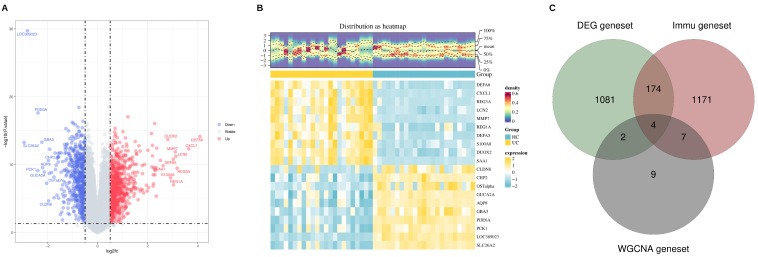
Bulk transcriptomic dysregulation and candidate gene intersection. (**A**) Volcano plot of UC-associated DEGs. The horizontal dashed line indicates the statistical significance threshold of *p* = 0.05, corresponding to −log10(*p*) = −log10(0.05), and the two vertical dashed lines indicate the log2 fold-change thresholds of −0.5 and 0.5. Genes satisfying |log2 fold change| > 0.5 and *p* < 0.05 were defined as DEGs. (**B**) Heatmap of DEGs. (**C**) Intersection of bulk DEGs, IICBRGs, and macrophage hdWGCNA blue module genes.

Bulk transcriptomic analysis in GSE179285 identified a broad mucosal disease signal. Using |log2 fold change| > 0.5 and *p* < 0.05, 1261 DEGs were detected between inflamed UC colon tissue and healthy control colon tissue. Among these genes, 719 were upregulated and 542 were downregulated in UC. The complete differential-expression results, including gene symbols, log2 fold changes, raw and adjusted p values, and regulation directions, are provided in Supplementary Materials, Additional File S2. The volcano plot showed the magnitude and significance of disease-associated expression changes, whereas the heatmap indicated that the DEGs captured a transcriptomic pattern distinguishing inflamed UC from healthy control mucosa (Figure 3A,B).

To link disease-level dysregulation with innate immune barrier biology and macrophage co-expression structure, three gene layers were intersected: UC-associated DEGs, the 1356-gene IICBRG set, and the 22-gene macrophage hdWGCNA blue module. This intersection yielded four candidate genes: CCL3, CCL4, JUNB, and FOS (Figure 3C). The candidate-gene intersection results are provided in Supplementary Materials, Additional File S1. The intersection strategy markedly reduced the broad UC transcriptomic signal and selected genes that simultaneously met disease association, innate immune barrier relevance, and macrophage co-expression criteria. This narrowed candidate set provided a focused basis for machine learning and downstream biological interpretation.

### 3.4. Machine Learning Retained CCL3, CCL4, and JUNB as Reproducible Macrophage-Associated Signatures

**Figure 4:**
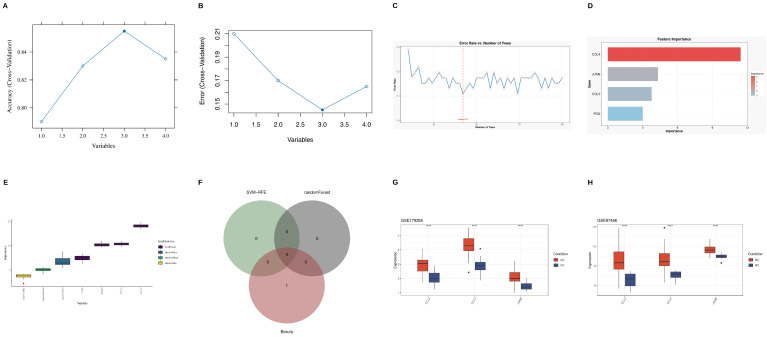
Machine learning and cross-dataset expression validation. (**A**,**B**) SVM-RFE accuracy and error curves. (**C**) Random forest tree selection based on the model error rate; ntree = 42 was selected according to the minimum error rate. (**D**) Random forest-based gene importance ranking of the candidate genes. (**E**) Boruta feature-selection output. (**F**) Feature prioritization intersection. (**G**) Discovery dataset expression of CCL3, CCL4, and JUNB. (**H**) Validation dataset expression of CCL3, CCL4, and JUNB. Statistical significance is indicated as follows: ****, *p* < 0.0001.

Three complementary feature prioritization approaches were applied to the four candidate genes in the discovery dataset. Support vector machine/recursive feature elimination selected CCL3, CCL4, and JUNB according to cross-validation accuracy and error patterns (Figure 4A,B). Random forest analysis evaluated tree numbers from 1 to 100, selected ntree = 42 based on the minimum error rate, and ranked CCL3, CCL4, and JUNB among the top features (Figure 4C,D). Boruta retained CCL3, CCL4, JUNB, and FOS as confirmed features (Figure 4E). The intersection of the three approaches retained CCL3, CCL4, and JUNB as the final macrophage-associated signatures (Figure 4F). The complete feature-selection outputs from SVM-RFE, random forest, and Boruta are provided in Supplementary Materials, Additional File S1.

Expression validation supported the reproducibility of these signatures across independent microarray platforms. In the GSE179285 discovery dataset, CCL3, CCL4, and JUNB were all significantly upregulated in inflamed UC samples compared with healthy controls. The same direction of expression change was reproduced in GSE87466, indicating that the upregulation pattern extended beyond the discovery platform (Figure 4G,H). Because CCL3 and CCL4 encode inflammatory chemokines and JUNB encodes a transcriptional regulator, the retained signature links chemokine-mediated inflammatory recruitment with broader transcriptional state regulation within UC mucosa.

### 3.5. CCL3, CCL4, and JUNB Showed Stable Transcriptomic Discriminative Capacity

**Figure 5:**
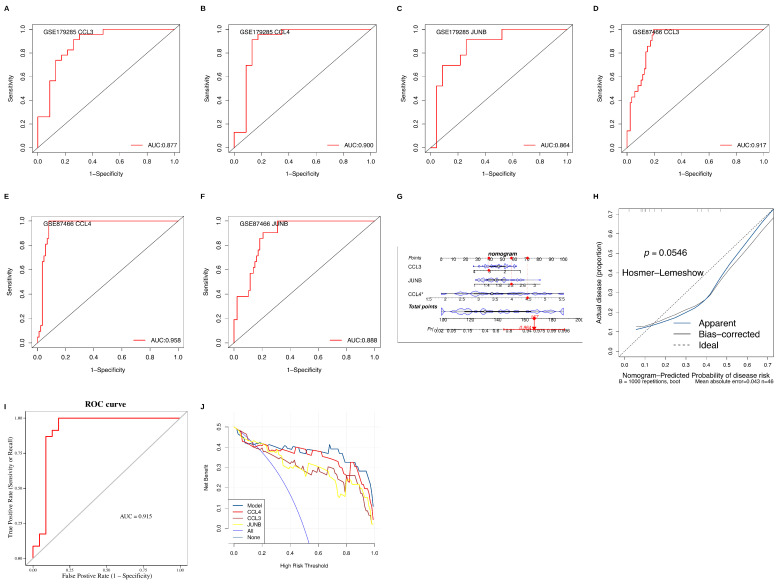
Receiver operating characteristic analysis and nomogram evaluation. (**A**–**C**) Discovery dataset Receiver operating characteristic (ROC) curves for CCL3, CCL4, and JUNB. (**D**–**F**) Validation dataset ROC curves for CCL3, CCL4, and JUNB. (**G**). Three-gene nomogram. (**H**) Calibration curve. (**I**) Combined ROC curve. (**J**) Decision curve analysis. Statistical significance is indicated as follows: *, 0.01 ≤ *p* < 0.05.

Receiver operating characteristic analysis showed that each final signature gene separated inflamed UC tissue from healthy control tissue in both datasets. In GSE179285, CCL3, CCL4, and JUNB achieved AUC values of 0.877, 0.900, and 0.864, respectively (Figure 5A–C). In GSE87466, the corresponding AUC values were 0.917, 0.958, and 0.888 (Figure 5D–F). CCL4 showed the highest individual AUC in both the discovery and validation datasets. These results indicate that the three genes retained consistent transcriptomic discrimination across independent cohorts and microarray platforms.

A three-gene nomogram was constructed in the discovery dataset to summarize the combined signature. The calibration curve showed acceptable agreement between predicted and observed probabilities, with a Hosmer–Lemeshow *p* value of 0.0546 (Figure 5G,H). The combined receiver operating characteristic curve yielded an AUC of 0.915 in the discovery dataset, and decision curve analysis indicated positive net benefit across a range of threshold probabilities (Figure 5I,J). These model summaries supported the combined transcriptomic signal of CCL3, CCL4, and JUNB while retaining the interpretation of the model as a retrospective discovery-stage expression signature.

### 3.6. Functional Enrichment and Localization Contextualized the Three-Gene Signature

**Figure 6:**
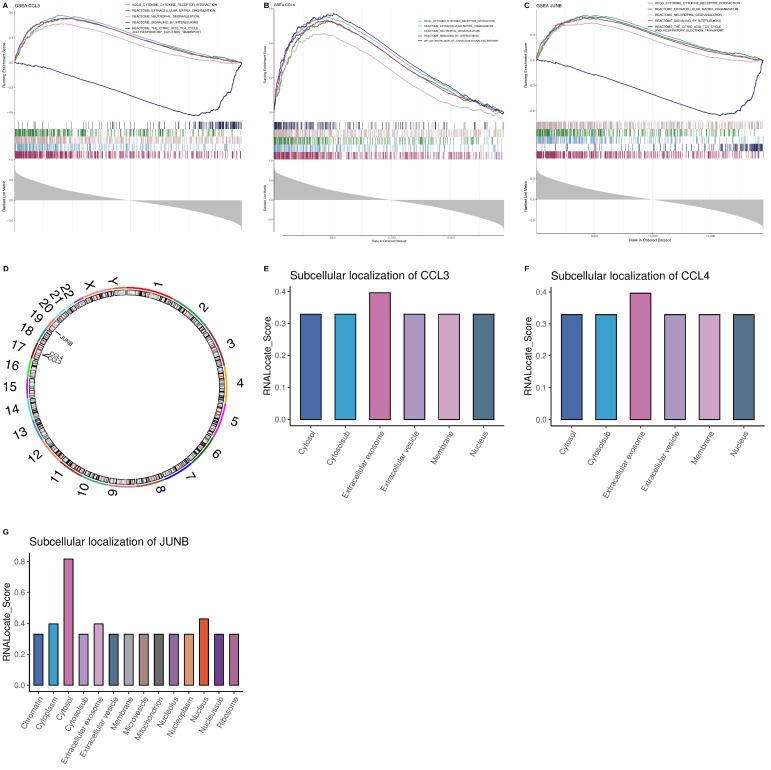
Functional enrichment and localization analysis. (**A**–**C**) GSEA plots for CCL3, CCL4, and JUNB-associated expression profiles. (**D**) Chromosomal localization of CCL3, CCL4, and JUNB. (**E**–**G**) Subcellular localization summaries for CCL3, CCL4, and JUNB.

Gene set enrichment analysis was performed for expression profiles associated with CCL3, CCL4, and JUNB. CCL3-associated ranking enriched 717 pathways, CCL4-associated ranking enriched 663 pathways, and JUNB-associated ranking enriched 727 pathways under the defined enrichment criteria. The complete GSEA results for the CCL3-, CCL4-, and JUNB-associated expression profiles are provided in Supplementary Materials, Additional File S3. The top visualized pathways for each gene reflected immune and inflammatory pathway programs (Figure 6A–C). These enrichment patterns were coherent with the known biological roles of CCL3 and CCL4 as chemokines and with the role of JUNB as a transcriptional component of inflammatory and stress response programs.

Chromosomal localization placed CCL3 at chromosome 17:36088256-36090169, CCL4 at chromosome 17:36103827-36105621, and JUNB at chromosome 19:12791486-12793315. The close chromosomal positioning of CCL3 and CCL4 is consistent with their related chemokine identity. RNALocate-based subcellular localization suggested extracellular exosome-related localization for CCL3 and CCL4 and cytosolic localization for JUNB (Figure 6D–G). These localization annotations provide descriptive molecular context. The secretory/extracellular localization pattern of CCL3 and CCL4 aligns with their chemokine function, whereas the JUNB localization pattern is compatible with broader intracellular regulatory activity.

### 3.7. Macrophage Re-Clustering and Pseudotime Associated the Signature with an Inflammatory State Continuum

**Figure 7:**
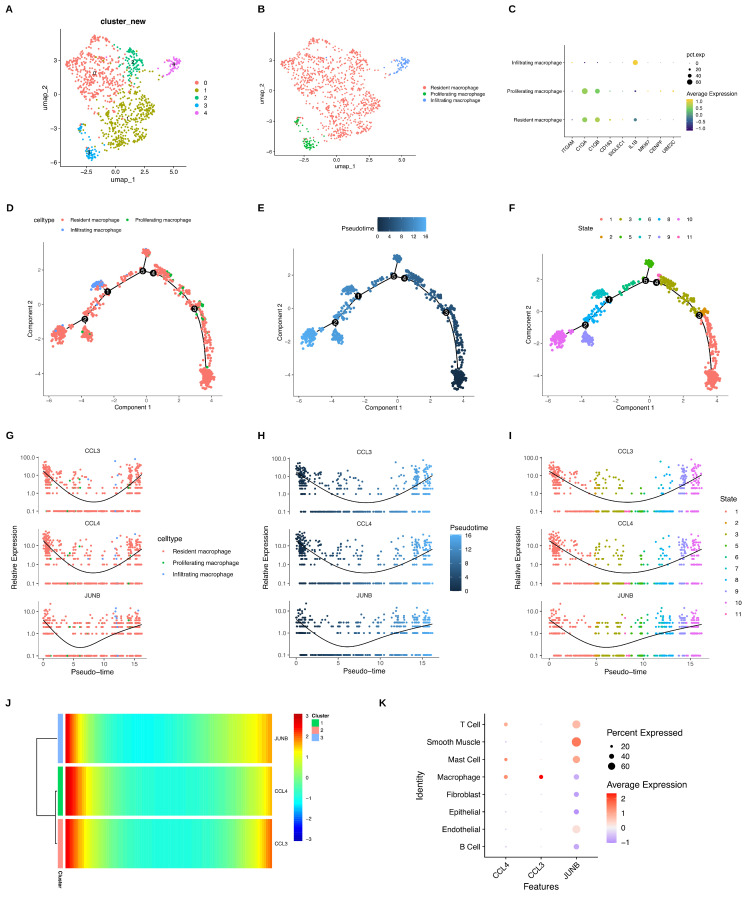
Macrophage re-clustering and pseudotime dynamics. (**A**) Macrophage UMAP clusters. (**B**) Annotated macrophage states. (**C**) Marker gene dot plot. (**D**–**F**) Pseudotime visualization by cell type, pseudotime value, and inferred state. The numbered nodes indicate key nodes or branch points along the inferred trajectory, representing computationally inferred positions at which marked changes or branching in macrophage transcriptional states may occur. (**G**–**I**) Pseudotime expression of CCL3, CCL4, and JUNB. (**J**) Pseudotime heatmap. (**K**) Single-cell dot plot for the final signatures.

Macrophages were extracted from the annotated single-cell atlas and re-clustered into five macrophage clusters. Marker gene patterns supported annotation into resident, proliferating, and infiltrating macrophage states (Figure 7A–C). This re-clustering provided a higher-resolution macrophage framework than the global cell-type annotation and allowed the final signatures to be interpreted within macrophage state organization. The state-focused design is consistent with current cell-resolved IBD studies, which emphasize that disease-related myeloid variation is often better captured at the state level than at the broad cell-type level.

Pseudotime ordering arranged macrophages along an inferred transcriptional state continuum. Visualization by macrophage state and pseudotime value showed that macrophage clusters formed a connected continuum of transcriptional remodeling (Figure 7D–F). CCL3, CCL4, and JUNB displayed dynamic expression along this continuum. CCL3 and CCL4 showed relatively high expression in early pseudotime regions, decreased during intermediate regions, and increased again in later regions. JUNB showed a similar broad dynamic pattern with reduced expression in the intermediate segment and re-elevation toward later pseudotime regions (Figure 7G–J). This high/low/high pattern suggests that the final signature marks macrophage transcriptional states with variable inflammatory activity rather than a uniformly expressed macrophage identity marker set.

The single-cell dot plot further supported macrophage-associated expression of CCL3 and CCL4, whereas JUNB showed a broader expression distribution across cell types while remaining relevant within the macrophage analysis framework (Figure 7K). This distinction is biologically important: CCL3 and CCL4 mainly represent inflammatory chemokine output, while JUNB may reflect a more general inflammatory transcriptional state that intersects with macrophage activation and antigen-related programs.

### 3.8. CellChat Analysis Indicated Disease State Remodeling of Macrophage-Centered Communication

**Figure 8:**
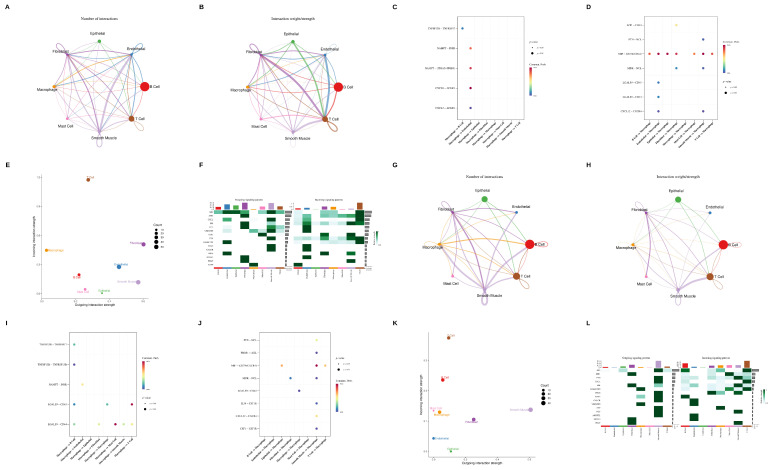
CellChat-based cell–cell communication inference in UC and healthy control samples. (**A**,**B**) UC interaction number and interaction strength networks. (**C**,**D**) UC macrophage ligand and macrophage receptor bubble plots. (**E**,**F**) UC outgoing and incoming signaling pattern summaries. (**G**,**H**) Healthy control interaction number and interaction strength networks. (**I**,**J**) Healthy control macrophage ligand and macrophage receptor bubble plots. (**K**,**L**) Healthy control signaling pattern summaries.

CellChat was applied separately to UC and healthy control cells to compare disease state communication structure. UC samples showed 123 inferred communication pathways, whereas healthy controls showed 81 pathways. This increase in inferred signaling diversity suggests that inflamed UC mucosa is associated with expanded intercellular communication complexity (Figure 8A,B,G,H). Such disease state rewiring is compatible with previous single-cell observations that IBD-associated tissue remodeling involves coordinated epithelial, stromal, endothelial, and myeloid interactions rather than isolated immune cell activation.

Macrophage-centered ligand analysis in UC identified prominent macrophage-to-endothelial CXCL8-ACKR1 signaling (Figure 8C). This interaction pattern is consistent with an inflamed mucosal environment in which macrophage-derived chemokine signaling may coordinate endothelial interaction and immune cell trafficking. Macrophage-centered receptor analysis in UC identified smooth muscle-to-macrophage MIF-(CD74 + CD44) signaling (Figure 8D), indicating that stromal and smooth muscle compartments may contribute incoming inflammatory cues to macrophages. UC outgoing and incoming signaling pattern summaries further supported heterogeneous macrophage-centered communication remodeling (Figure 8E,F).

Healthy control samples displayed a different communication structure. The strongest macrophage outgoing signal in controls involved macrophage-to-mast-cell LGALS9-CD44 signaling, while smooth muscle-to-macrophage MIF-(CD74 + CD44) signaling remained prominent as an incoming interaction (Figure 8I,J). The difference between UC and healthy control outputs suggests a shift in macrophage communication partners and ligand/receptor usage during mucosal inflammation. In this context, the CCL3/CCL4/JUNB signature can be interpreted as marking macrophage inflammatory states embedded within broader disease-remodeled cell–cell communication networks.

### 3.9. Virtual Perturbation Connected CCL3, CCL4, and JUNB to Distinct Macrophage Programs

**Figure 9:**
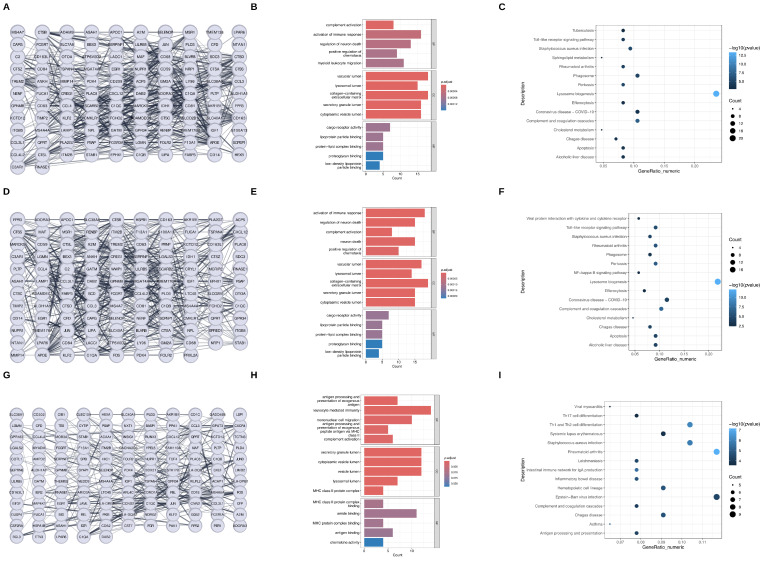
Virtual perturbation and enrichment analysis. (**A**,**D**,**G**) STRING-based PPI networks for CCL3, CCL4, and JUNB perturbation outputs. (**B**,**E**,**H**) Gene ontology enrichment summaries. (**C**,**F**,**I**) KEGG enrichment summaries.

Virtual gene perturbation in macrophages identified 112 perturbed genes for CCL3, 118 perturbed genes for CCL4, and 127 perturbed genes for JUNB. The complete virtual-perturbation gene lists and the corresponding Gene Ontology and KEGG enrichment results are provided in Supplementary Materials, Additional File S3. STRING-based PPI networks summarized the corresponding perturbation outputs. The CCL3 network contained 112 nodes and 477 edges; the CCL4 network contained 118 nodes and 468 edges; and the JUNB network contained 126 nodes and 235 edges because one perturbed gene was not mapped in the STRING Homo sapiens database (Figure 9A,D,G).

The CCL3 perturbation output was enriched for biological processes including complement activation, activation of immune response, positive regulation of chemotaxis, and myeloid leukocyte migration. Cellular component terms included vacuolar lumen, lysosomal lumen, secretory granule lumen, cytoplasmic vesicle lumen, and collagen-containing extracellular matrix. Molecular function terms included cargo receptor activity, lipoprotein particle binding, protein–lipid complex binding, proteoglycan binding, and low-density lipoprotein particle binding (Figure 9B). KEGG enrichment linked the CCL3 perturbation output to lysosome-associated programs (Figure 9C).

The CCL4 perturbation output showed a closely related enrichment pattern. Biological processes included activation of immune response, complement activation, positive regulation of chemotaxis, and related inflammatory programs. Cellular component enrichment again highlighted vacuolar lumen, lysosomal lumen, secretory granule lumen, cytoplasmic vesicle lumen, and collagen-containing extracellular matrix. Molecular function terms overlapped with CCL3-associated outputs and included cargo receptor activity, lipoprotein particle binding, protein–lipid complex binding, proteoglycan binding, and low-density lipoprotein particle binding (Figure 9E). KEGG enrichment again pointed to lysosome-related macrophage programs (Figure 9F). This convergence between CCL3 and CCL4 supports a shared chemokine-linked perturbation axis involving immune activation, vesicular compartments, and leukocyte migration.

JUNB perturbation produced a different functional profile. Biological processes were concentrated in antigen processing and presentation of exogenous antigen, leukocyte-mediated immunity, mononuclear cell migration, antigen processing and presentation of exogenous peptide antigen via major histocompatibility complex (MHC) class II, and complement activation. Cellular component terms included secretory granule lumen, cytoplasmic vesicle lumen, vesicle lumen, lysosomal lumen, and MHC class II protein complex. Molecular function terms included MHC class II protein complex binding, MHC protein complex binding, antigen binding, amide binding, and chemokine activity (Figure 9H). KEGG enrichment associated the JUNB perturbation output with immune disease-related pathways (Figure 9I). These results distinguish JUNB from CCL3 and CCL4 by linking it more strongly to antigen presentation and MHC class II-related macrophage programs.

### 3.10. Transcription Factor Activity, Compound Prioritization, and Docking Extended the Network-Level Interpretation

**Figure 10:**
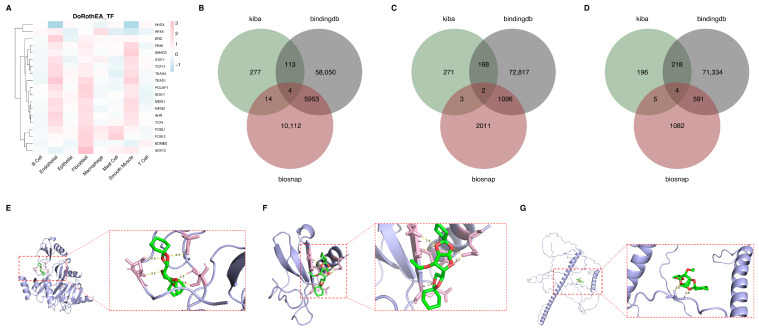
Transcription factor prioritization, GraphBAN compound prediction, and molecular docking. (**A**) Macrophage transcription factor activity heatmap. (**B**–**D**) GraphBAN intersection plots for CCL3, CCL4, and JUNB. (**E**–**G**) Molecular docking poses of CID11879209 with CCL3, CCL4, and JUNB. ADDIN NE.Bib.

Transcription factor activity analysis prioritized RFX5 as the highest-scoring transcription factor in macrophages (Figure 10A). Because RFX5 is associated with MHC class II transcriptional regulation, its prioritization is coherent with the JUNB perturbation output enriched for antigen-processing and presentation-related terms. This result adds an upstream regulatory layer to the macrophage-centered signature interpretation.

GraphBAN compound protein interaction prediction identified predicted compounds for the final signatures. CCL3 was associated with four predicted compounds, CCL4 with two predicted compounds, and one shared candidate, PubChem CID11879209, overlapped across CCL3, CCL4, and JUNB prediction outputs (Figure 10B–D). The complete GraphBAN compound-prediction results for CCL3, CCL4, and JUNB are provided in Supplementary Materials, Additional File S3. Molecular docking then visualized predicted interaction poses between CID11879209 and each signature protein. The predicted binding energies were −6.55 kcal/mol for CCL3, −6.31 kcal/mol for CCL4, and −4.50 kcal/mol for JUNB (Figure 10E–G). The stronger docking scores for CCL3 and CCL4 than for JUNB are compatible with the chemokine-centered component of the signature. These compound and docking results provide a ranked computational hypothesis for future pharmacological evaluation.

## 4. Discussion

This study identified CCL3, CCL4, and JUNB as macrophage-associated inflammatory regulatory signatures in UC through integration of bulk transcriptomic microarray data, single-cell RNA-seq, innate immune cell barrier gene scoring, macrophage co-expression analysis, machine learning, cross-dataset validation, macrophage trajectory inference, intercellular communication analysis, virtual perturbation, transcription factor activity analysis, and compound-prioritization modeling. The principal contribution is the construction of a cell-type-aware interpretation of a compact inflammatory signature. The final genes were supported by disease-level differential expression, innate immune barrier annotation, macrophage co-expression structure, reproducible expression upregulation, single-cell state localization, and network-level functional interpretation. This is aligned with the current view that UC is a heterogeneous inflammatory disorder in which tissue-level disease activity reflects interacting epithelial, stromal, vascular, and immune cell programs rather than a single linear pathway [38].

Macrophages are a biologically coherent focus for this analysis. In intestinal tissue, macrophages contribute to microbial recognition, cytokine production, chemokine release, phagocytosis, antigen presentation, epithelial injury, matrix remodeling, and repair processes. Recent reviews emphasize that intestinal macrophages are highly plastic cells whose phenotypes are shaped by tissue niche, ontogeny, microbial exposure, inflammatory cues, and disease stage [39,40]. In active UC tissue, macrophage states are influenced by epithelial damage, stromal signals, microbial products, and cytokine gradients, and recent UC-focused reviews have highlighted macrophage polarization and therapeutic targeting as important mechanistic themes [41]. In the present analysis, AUCell prioritized macrophages among innate immune candidate populations, and hdWGCNA identified a macrophage blue module that overlapped with UC bulk DEGs and IICBRGs. This multi-layer support strengthens the interpretation that the final genes reflect macrophage-linked inflammatory biology rather than nonspecific tissue inflammation.

CCL3 and CCL4 formed the chemokine arm of the signature. Both genes were upregulated in discovery and validation datasets, were embedded in the macrophage-centered co-expression module, and showed dynamic macrophage pseudotime patterns. Their virtual perturbation outputs converged on complement activation, immune response activation, chemotaxis, myeloid leukocyte migration, vacuolar and lysosomal lumen annotations, secretory granule lumen, and protein–lipid or lipoprotein particle binding. Chemokine biology is particularly relevant in intestinal inflammation because chemokine gradients regulate leukocyte recruitment, endothelial interaction, and inflammatory cell positioning; CXCL8 biology, for example, has been extensively linked with neutrophil recruitment and pharmacological targeting efforts [42,43]. CCL3 has also emerged in machine learning-based UC biomarker studies, while CCL4-based targeting strategies have shown anti-inflammatory effects in experimental intestinal inflammation models [44,45]. These data support interpretation of CCL3 and CCL4 as markers of macrophage states involved in inflammatory recruitment, vesicular processing, and leukocyte trafficking.

The CCL3/CCL4 perturbation results also connect the signature with lysosomal and vesicular macrophage programs. Vesicular trafficking, phagolysosomal processing, antigen handling, and inflammatory mediator release are central macrophage functions in the gut [39,40]. In this study, both CCL3 and CCL4 perturbation outputs enriched vacuolar lumen, lysosomal lumen, secretory granule lumen, and cytoplasmic vesicle lumen annotations, suggesting that the chemokine component of the signature is coupled with intracellular processing programs rather than only extracellular chemokine signaling. This interpretation is consistent with prior UC computational studies that identified lysosomal autophagy-related hub genes and experimentally supported lysosome-associated mechanisms in UC tissue [11].

JUNB provided a complementary transcriptional state component. Unlike CCL3 and CCL4, JUNB showed broader expression across multiple cell types, and it should therefore be interpreted as macrophage-relevant rather than macrophage-specific. Its perturbation output was enriched for antigen processing and presentation of exogenous antigen, MHC class II-related annotations, leukocyte-mediated immunity, mononuclear cell migration, and immune disease-related pathways. JUNB is an AP-1 family transcription factor involved in immune regulation, inflammatory responses, and microenvironmental remodeling [46]. The antigen presentation signal is also biologically plausible because MHC class II antigen presentation is a central regulatory feature of mucosal immune responses, and recent work has shown that intestinal epithelial MHC class II presentation can tune bacteria-reactive CD4 T cell responses [47]. Broader MHC class I and class II biology further supports the relevance of polymorphism, peptide loading, and antigen presentation in shaping adaptive immune activation [48]. In UC, JUNB may therefore reflect a transcriptional activation program that intersects with macrophage antigen presentation and inflammatory remodeling.

The pseudotime analysis added a dynamic single-cell layer to the bulk-derived signature. CCL3, CCL4, and JUNB showed an early/high, middle/low, and late-reinforced pattern along the inferred macrophage transcriptional state continuum. This pattern suggests that the signature is not simply a fixed macrophage identity marker set. Instead, it may capture state-dependent inflammatory activity within macrophage remodeling. Longitudinal and treatment-linked single-cell studies in IBD show that myeloid and mononuclear phagocyte states can change with therapy and disease context [49,50]. Because the single-cell dataset analyzed here is cross-sectional, pseudotime should be interpreted as an inferred transcriptional continuum rather than chronological disease progression. Even under this constraint, the trajectory result provides useful context by showing that the three genes vary coherently across macrophage state organization.

CellChat analysis placed the signature within disease state communication remodeling. UC samples exhibited more inferred communication pathways than healthy controls, and the macrophage-centered interaction pattern differed by disease state. The UC macrophage-to-endothelial CXCL8-ACKR1 signal suggests potential coordination between inflammatory myeloid states and endothelial trafficking functions. This interpretation is supported by experimental work showing that macrophage/endothelial cell crosstalk can orchestrate neutrophil recruitment in inflamed mucosa [51]. In parallel, vedolizumab response studies have shown that clinical response in UC can be associated with altered immune cell–cell communication networks [52], and single-cell/spatial multi-omics analyses have highlighted therapy-related alterations across cellular compartments in UC [50]. The smooth muscle-to-macrophage MIF-(CD74 + CD44) signal observed here further suggests that stromal and smooth muscle compartments may provide incoming inflammatory cues to macrophages. These inferred interactions do not establish direct regulation by CCL3, CCL4, or JUNB, but they locate the final signature within macrophage states embedded in a remodeled mucosal communication network.

The transcriptomic discrimination results add another layer of support. CCL3, CCL4, and JUNB were consistently upregulated in two independent bulk microarray datasets and achieved validation AUC values of 0.917, 0.958, and 0.888, respectively. CCL4 showed the highest single-gene AUC in both the discovery and validation datasets. The three-gene nomogram summarized the combined behavior of these signatures in the discovery dataset, with a combined AUC of 0.915 and acceptable calibration. Molecular signatures in UC are clinically relevant because current therapeutic decisions still face challenges related to disease heterogeneity, treatment positioning, and incomplete mucosal healing. However, these results should be interpreted as robust retrospective discrimination between inflamed UC and healthy control tissue and as support for further evaluation in relation to disease activity, severity, treatment response, and tissue healing.

The interpretation of CCL3, CCL4, and JUNB differs from a conventional hub gene list because each gene is connected to a distinct evidence layer. CCL3 and CCL4 carry chemokine and vesicular/lysosomal signatures; JUNB carries a transcriptional and antigen presentation signal; all three are reproducibly upregulated in UC tissue; and all three are linked to macrophage state organization. Previous integrated UC studies have prioritized lysosomal autophagy-associated genes or aryl hydrocarbon receptor-associated genes through multi-step bioinformatics analysis and validation [11,12]. Other machine learning and multi-microarray studies have similarly shown that transcriptomic feature selection can identify reproducible UC-associated gene signatures, although the biological value of such signatures depends on cell-type context and independent validation [44]. The present study extends this computational framework toward a macrophage-centered inflammatory regulatory axis and emphasizes cell state interpretation of bulk transcriptomic signals.

The regulatory and compound prioritization analyses should be viewed as extensions of biological interpretation. RFX5 prioritization was coherent with the antigen-presentation and MHC class II signal observed in the JUNB perturbation output. This regulatory layer is consistent with the role of MHC class II transcriptional regulation in mucosal antigen presentation [47,48]. GraphBAN and docking nominated CID11879209 as a shared computational compound candidate, with stronger predicted docking support for CCL3 and CCL4 than for JUNB. Related work on CCL4-mediated targeting of spleen tyrosine kinase inhibition in intestinal inflammation supports the broader concept that chemokine-linked delivery or targeting strategies may have translational relevance [45]. Nevertheless, docking scores provide a structural compatibility estimate under simplified assumptions and do not establish binding affinity, target engagement, pharmacodynamics, pharmacokinetics, safety, or disease-modifying activity.

Several limitations should be considered. First, this study used retrospective public datasets, and the bulk datasets were microarray-based rather than RNA sequencing-based. Probe annotation, platform differences, sample handling, and cohort composition may influence expression-level comparisons. Second, the single-cell dataset provided valuable cell-type resolution but included a limited number of donors, making donor-level heterogeneity a potential source of variation. Third, the nomogram and decision curve analysis were generated from the discovery dataset and should be interpreted as exploratory model summaries. Fourth, AUCell, hdWGCNA, CellChat, scTenifoldKnk, DoRothEA, GraphBAN, and docking all rely on computational assumptions and should be interpreted within their methodological constraints. Fifth, the present findings require prospective tissue-level validation, protein-level validation, spatial localization, and functional studies to determine whether the signatures are biomarkers, mechanistic contributors, or both.

Despite these limitations, this study provides a coherent macrophage-centered transcriptomic framework for UC. By integrating bulk disease signals with single-cell macrophage state organization and regulatory network interpretation, the analysis refines CCL3, CCL4, and JUNB from DEGs into a biologically contextualized inflammatory signature. This framework may help guide future studies that combine clinical metadata, longitudinal sampling, spatial profiling, and functional perturbation to clarify how macrophage-centered inflammatory programs contribute to UC activity and tissue remodeling.

## 5. Conclusions

This study identified CCL3, CCL4, and JUNB as macrophage-associated inflammatory regulatory signatures in UC by integrating public bulk transcriptomic microarray data with single-cell RNA-seq-based macrophage interpretation. The final signatures were supported by disease-level differential expression, innate immune barrier gene annotation, macrophage co-expression structure, reproducible expression upregulation, and stable transcriptomic discrimination between inflamed UC and healthy control tissue. Single-cell trajectory analysis, cell–cell communication inference, virtual perturbation, and transcription factor activity analysis further placed these genes within macrophage state remodeling, disease state intercellular communication, chemotaxis, lysosome-associated programs, antigen processing and presentation, and candidate upstream regulatory activity.

CCL3 and CCL4 primarily represent a chemokine-centered inflammatory component linked to macrophage chemotaxis, immune activation, vesicular compartments, and lysosome-related processes. JUNB represents a broader inflammatory transcriptional state component associated with antigen presentation and MHC class II-related programs. Together, these genes define a compact macrophage-centered signature that connects tissue-level UC dysregulation with cell state-specific inflammatory remodeling.

The findings support further validation of CCL3, CCL4, and JUNB in independent UC cohorts, protein-level assays, spatial tissue profiling, and functional macrophage models. The compound prioritization results nominate CID11879209 as a computational lead for future evaluation.

## Data Availability

The datasets analyzed during the current study are available in GEO (https://www.ncbi.nlm.nih.gov/geo/ accessed on 30 April 2026) under accession numbers GSE179285, GSE87466, and GSE231993.

## Supplementary Materials

The following supporting information can be downloaded at https://www.mdpi.com/article/10.3390/genes17070841/s1, Additional File S1. XLSX. Macrophage hdWGCNA blue module genes, candidate gene intersection results, and feature prioritization outputs for SVM-RFE, random forest, and Boruta. Additional File S2. XLSX. Full list of DEGs from the GSE179285 UC versus healthy control analysis, including gene symbol, log2 fold change, *p* value, adjusted *p* value if available, and regulation direction. Additional File S3. XLSX. Full GSEA, virtual perturbation, and GraphBAN compound prediction outputs for CCL3, CCL4, and JUNB.

## Abbreviations

AUC	Area under the curve
AUCell	Area under the recovery curve-based single-cell gene-set scoring
DEG	Differentially expressed gene
GEO	Gene Expression Omnibus
hdWGCNA	high-dimensional Weighted Gene Co-Expression Network Analysis
IBD	Inflammatory bowel disease
IICBRG	Innate immune cell barrier-related gene
KEGG	Kyoto Encyclopedia of Genes and Genomes
MHC	Major histocompatibility complex
MSigDB	Molecular Signatures Database
PCA	Principal component analysis
PPI	Protein–protein interaction
ROC	Receiver operating characteristic
STRING	Search Tool for the Retrieval of Interacting Genes/Proteins
UC	Ulcerative colitis
UMAP	Uniform manifold approximation and projection
